# Item-Level Story Recall Predictors of Amyloid-Beta in Late Middle-Aged Adults at Increased Risk for Alzheimer’s Disease

**DOI:** 10.3389/fpsyg.2022.908651

**Published:** 2022-06-27

**Authors:** Kimberly D. Mueller, Lianlian Du, Davide Bruno, Tobey Betthauser, Bradley Christian, Sterling Johnson, Bruce Hermann, Rebecca Langhough Koscik

**Affiliations:** ^1^Department of Communication Sciences and Disorders, University of Wisconsin-Madison, Madison, WI, United States; ^2^Department of Biostatistics and Medical Informatics, School of Medicine and Public Health, University of Wisconsin-Madison, Madison, WI, United States; ^3^Wisconsin Alzheimer’s Disease Research Center, School of Medicine and Public Health, University of Wisconsin-Madison, Madison, WI, United States; ^4^School of Psychology, Liverpool John Moores University, Liverpool, United Kingdom; ^5^Waisman Laboratory for Brain Imaging and Behavior, University of Wisconsin-Madison, Madison, WI, United States; ^6^Department of Medical Physics, University of Wisconsin-Madison, Madison, WI, United States; ^7^Geriatric Research Education and Clinical Center, William S. Middleton Veterans Hospital, Madison, WI, United States; ^8^Department of Neurology, School of Medicine and Public Health, University of Wisconsin-Madison, Madison, WI, United States

**Keywords:** Alzheimer’s disease, mild cognitive impairment, language, dementia, positron emission tomography, amyloid beta, cognitive decline and dementia

## Abstract

**Background:**

Story recall (SR) tests have shown variable sensitivity to rate of cognitive decline in individuals with Alzheimer’s disease (AD) biomarkers. Although SR tasks are typically scored by obtaining a sum of items recalled, item-level analyses may provide additional sensitivity to change and AD processes. Here, we examined the difficulty and discrimination indices of each item from the Logical Memory (LM) SR task, and determined if these metrics differed by recall conditions, story version (A vs. B), lexical categories, serial position, and amyloid status.

**Methods:**

*n* = 1,141 participants from the Wisconsin Registry for Alzheimer’s Prevention longitudinal study who had item-level data were included in these analyses, as well as a subset of *n* = 338 who also had amyloid positron emission tomography (PET) imaging. LM data were categorized into four lexical categories (proper names, verbs, numbers, and “other”), and by serial position (primacy, middle, and recency). We calculated difficulty and discriminability/memorability by item, category, and serial position and ran separate repeated measures ANOVAs for each recall condition, lexical category, and serial position. For the subset with amyloid imaging, we used a two-sample *t*-test to examine whether amyloid positive (Aβ+) and amyloid negative (Aβ−) groups differed in difficulty or discrimination for the same summary metrics.

**Results:**

In the larger sample, items were more difficult (less memorable) in the delayed recall condition across both story A and story B. Item discrimination was higher at delayed than immediate recall, and proper names had better discrimination than any of the other lexical categories or serial position groups. In the subsample with amyloid PET imaging, proper names were more difficult for Aβ+ than Aβ−; items in the verb and “other” lexical categories and all serial positions from delayed recall were more discriminate for the Aβ+ group compared to the Aβ− group.

**Conclusion:**

This study provides empirical evidence that both LM stories are effective at discriminating ability levels and amyloid status, and that individual items vary in difficulty and discrimination by amyloid status, while total scores do not. These results can be informative for the future development of sensitive tasks or composite scores for early detection of cognitive decline.

## Introduction

Alzheimer’s disease research studies are increasingly focused on identifying those participants who are at the earliest stages on the continuum of Alzheimer’s disease (AD), when AD pathology is present but cognitive decline is subtle or absent (Arenaza-Urquijo and Vemuri, 2018). It is during this timeframe when treatments are likely to show the most benefit in slowing or preventing AD clinical signs and symptoms (Food and Drug Administration, 2018). To this end, it is important to identify cognitive measures that are highly sensitive to cognitive decline at the preclinical phase. Most long-standing neuropsychological tests used in AD studies were originally designed to detect decline associated with Mild Cognitive Impairment (MCI, often the precursor to dementia) or dementia, but are often insensitive to subtle changes associated with AD pathology when overt symptoms may not be present, but still fall within the normative range (i.e., “preclinical AD”; Mortamais et al., 2017; Jutten et al., 2021). The National Institute on Aging - Alzheimer’s Association (NIA-AA) research framework for Alzheimer’s disease defines this as Stage 2, when cognitive decline may be documented by evidence of subtle decline on longitudinal testing, subjective cognitive complaints, or both (Jessen et al., 2014, 2020; Jack et al., 2018).

Performance on commonly utilized neuropsychological tests is typically described and analyzed by calculating an aggregate of correctly recalled or answered items into a total score. This is true for tests of episodic memory, such as word list learning and memory [e.g., Rey Auditory Verbal Learning Test (R-AVLT); Schmidt, 1996] and non-verbal figure learning and memory [e.g., Brief Visuospatial Memory Test (BVMT); Benedict et al., 1996], as well as for tests of semantic memory such as category fluency tests (e.g., “name as many animals as you can think of in 60 s”) or confrontation naming tasks (e.g., Boston Naming Test; Goodglass and Kaplan, 1983). However, multiple studies have shown that detailed, item-level analyses of these data can provide additional information that is either more sensitive than the total score alone, informative about the underlying mechanisms of task performance in both disease and typical aging, or both. For example, while impairment in category fluency tasks (as measured by total score) is a well-known distinguishing factor between dementia, MCI, and typical aging (Putcha et al., 2020), the mechanisms of this impairment and whether or not the difficulty stems from degradation of the semantic store (i.e., temporal lobe memory functions), or from search and selection retrieval processes (i.e., frontal lobe executive control processes), is under investigation through item-level analyses (Weakley and Schmitter-Edgecombe, 2014; Papp et al., 2016, 2017). Specifically, in category fluency tasks, the kinds of words recalled are analyzed according to subcategories (“clusters”), and the temporal processes of moving from one cluster to the next are referred to as “switches,” with the latter representing the executive control portion of the task and cluster size representing the semantic storage component (Troyer et al., 1998). Other item-level approaches to memory and language testing include measuring the serial position effect in list learning tasks (Bruno et al., 2016, 2018), or analyzing the types of cues needed for naming tasks (phonemic vs. semantic cues; Balthazar et al., 2008; Lin et al., 2014), all with the goal of understanding the basis of dysfunction. A potential primary endpoint for these item-level approaches is the development of more sensitive measures for early detection of cognitive decline based on the patterns of neuropathology and their associated functions.

Recently our group deconstructed another commonly utilized episodic memory test for early detection of decline due to AD: the story recall task, “Logical Memory” from the Wechsler Memory Scale-Revised, stories A and B (WMS-R; Wechsler, 1987). In this task, the participant listens to a story read aloud and is instructed to “tell me everything I read to you, using as close to the same words as you can, begin at the beginning,” immediately after hearing the story, and again after a 30-min delay. In our first paper (Mueller et al., 2020), we examined whether recall of items from stories A and B that belonged to a particular lexical category (proper names, verbs, or numerical expressions) was more likely to be associated with cognitively unimpaired participants at substantially higher risk of AD dementia due to positivity for amyloid-beta (Aβ+) vs. those who were amyloid negative (Aβ−). We found a compelling association between Aβ+ and proper names, such that participants who were Aβ+ were less likely to recall proper names (across stories A and B) at the 30-min delay than those who were Aβ−. We did not find this association with the total score. Interestingly, the two groups did not differ on proper name recall at the immediate delay condition, suggesting a deficit with retrieval and/or storage, but not learning.

Another prior study using data from this cohort examined item-level data from Logical Memory to determine if the serial position of the items’ presentation was associated with progression to clinical MCI or with Aβ+/−. In typical aging, items at the beginning of the list (i.e., primacy items) and items at the end of the list (i.e., recency items) are recalled more easily than items in the middle, but in persons with MCI and dementia, recall of the primacy items tends to be poorer (La Rue et al., 2008; Bruno et al., 2013; Talamonti et al., 2020), and there is a prominent loss of recency recall between immediate and delayed testing (Bruno et al., 2016, 2018). In this second study, we calculated serial position (primacy, middle, and recency; i.e., the end of the story) effects in the Logical Memory story and found a loss of recall for the primacy items from immediate to delayed recall in individuals who progressed to Aβ+ status (Bruno et al., 2020).

Although evidence shows that there is similar sensitivity and specificity in both immediate and delayed recall conditions in discriminating between dementia, MCI, and healthy controls, this prior research evaluated total scores (Weissberger et al., 2017). Similarly, even in nonverbal tasks, participants with AD dementia performed worse on immediate, delayed and recognition tasks than healthy controls or participants with depression (Contador et al., 2010). Furthermore, there is controversy regarding whether rates of encoding (learning) vs. disrupted storage of learned material are the primary deficit in AD dementia (Christensen et al., 1998). This and other previous research have involved patients with clinical impairment (i.e., dementia), and many of these studies have evaluated aggregated scores as opposed to item-level or process scores. It is largely unknown how these memory processes are affected very early in the disease continuum (i.e., at the stage when AD neuropathology is developing but cognition is not clinically impaired, or “preclinical AD”). It is possible that item-level analyses allow for more fine-grained understanding of early cognitive changes.

Neural correlates and neural network theories are compelling explanations as to why we saw a proper name effect in persons who were Aβ+: first, proper name recall has been localized to the inferior anterior temporal lobe (Ross et al., 2010; Semenza, 2011; Fresnoza et al., 2022), adjacent to regions such as the perirhinal and entorhinal cortices, which are sites of early AD neuropathology accumulation (Braak et al., 2011). Second, the neural networks (attributes and similarities that aid in recall) are sparse for names of people and places compared to regular nouns. However, a potential confound exists, in that the Logical Memory task has a high concentration of proper names at the beginning of the two stories (story A and story B). Thus, the need to disambiguate proper name effects from their position in the story is important for understanding the mechanistic principles underlying deficits in story recall due to ADRD. One method for understanding contributing factors to disparate performance on proper name recall between Aβ groups is by examining the item-level difficulty, as was done by Salthouse (2017). In that study, item recall patterns were compared across differing age groups, differing baseline memory ability groups, and groups showing longitudinal decline. The study found uniform differences in item difficulty across age, ability and longitudinal decline groups. The study also included memorability analyses across different serial positions, in which item accuracy in the poorer-performing group was plotted as a function of item accuracy in the better-performing group.

Results showed lower memorability of items in the primacy and recency positions for delayed recall than for immediate recall (Salthouse, 2017). Whether item-level difficulty patterns from story recall differ between groups at increased/decreased risk for Alzheimer’s disease is unknown and has the potential to provide information about sensitive measures for AD-related cognitive decline. By identifying specific items or groups of items that are most sensitive to AD-related decline, shortened versions of tests or automated scoring algorithms can be developed for screening, early detection, and disease monitoring.

The present study had two aims: first, using a large sample of late-middle-aged adults from the Wisconsin Registry for Alzheimer’s Prevention (WRAP; *n* = 1,141, cognitively unimpaired at baseline), we calculated difficulty and discrimination indices of each item by study visit and recall condition (immediate and delayed) from the Logical Memory story recall task. We then examined whether these metrics differed between recall conditions, story versions (stories A vs. B), lexical categories, or serial position groups. For the second aim, we used the subset that had completed positron emission tomography (PET) amyloid imaging (*n* = 338) and calculated difficulty and discrimination indices separately for the Aβ+ (*n* = 79) and Aβ− (*n* = 259) groups. We then examined whether these metrics differed between Aβ+ and Aβ− groups by recall condition, story version, lexical categories, and serial position groups.

## Materials and Methods

### Participants

Participants were drawn from WRAP, a longitudinal cohort study enriched for parental history of late-onset sporadic AD (Sager et al., 2005; Johnson et al., 2018). WRAP visits began in 2001; participants are excluded from enrollment if they have a prior diagnosis of dementia or evidence of dementia at baseline testing. The baseline mean age is 54 years, 73% have a parent with AD dementia, and 40% of the total sample are *APOE* ε4 carriers. Participants complete detailed neuropsychological testing, medical examinations, and health and lifestyle questionnaires at each biennial visit (*n* = 1778, range of visits = 1–7). To track subtle, preclinical and/or clinically significant decline, WRAP researchers developed a “robust” norms approach in which internal normative distributions for cognitive test scores are generated adjusting for age, sex, and literacy, where the normative group is non-declining over time. An algorithm was created according to the robust norms to “flag” participants who are declining outside the range of the internal norms (1.5 SDs below the robust normative means). The flagged participants’ cognitive test performance, medical history, subjective and informant appraisals of memory, and medical examinations are reviewed and one of four determinations of cognitive status are made, based on NI Aβ-AA criteria (Albert et al., 2011; McKhann et al., 2011; Jack et al., 2018): “cognitively unimpaired—stable,” “cognitively unimpaired—declining,” “MCI,” “Impaired not MCI,” or “dementia.” Further details regarding these approaches are detailed elsewhere (Koscik et al., 2014, 2019; Clark et al., 2016; Jonaitis et al., 2019; Langhough Koscik et al., 2021).

**Figure 1:**
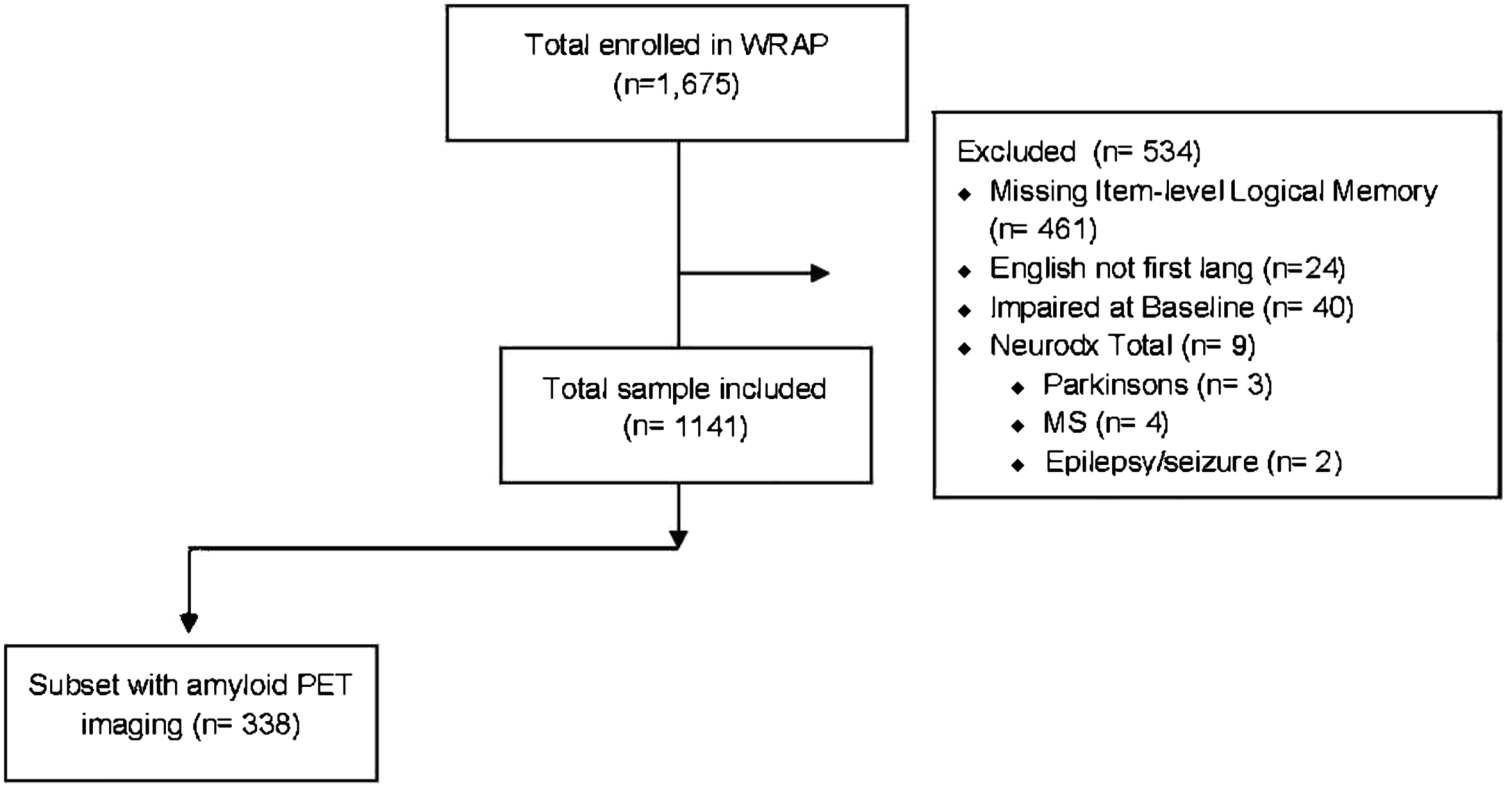
Flowchart indicating the study analysis inclusion/exclusion criteria applied to the Wisconsin Registry for Alzheimer’s Prevention longitudinal cohort.

Participants were included in the present study if they were native English speakers, had complete item level data from the Logical Memory test for at least one visit, were clinically unimpaired (no diagnosis of MCI or dementia) at their baseline Logical Memory visit (median = visit 2), were free from neurological disorders at any visit including Parkinson’s disease, multiple sclerosis, stroke, or epilepsy/seizures (Figure 1; *n* = 1,141). A subset of participants who had completed amyloid PET scans (completed near WRAP visit median = 3) and met the above-described inclusion criteria (*n* = 338) were used for the second aim. All activities for this study were approved by the University of Wisconsin-Madison Institutional Review Board and completed in accordance with the Declaration of Helsinki.

### Items and Variables From Logical Memory Story Recall

Logical Memory is a story recall subtest from the WMS-R (Wechsler, 1987), a standardized, norm-referenced assessment of learning and episodic memory. Logical Memory was introduced to the WRAP battery at the median visit 2; thus, “baseline” in the present study refers to each participant’s first Logical Memory assessment. Standardized test administration procedures for both stories A and B were followed in accordance with the WMS-R manual. Participants were read the following instructions prior to reading each story verbatim: “I am going to read you a story of just a few lines, and when I am through, tell the story back to me, using as close to the same words as you can remember; you should tell me all you can remember, even if you are not sure.” Participants immediately recalled each story following presentation (immediate recall) and again after a 25–35-min delay (delayed recall). The traditional scoring procedure includes 25 items or “idea units,” which comprise the item-level data used for these analyses. For the lexical categories which are described in detail elsewhere (Mueller et al., 2020), we assigned idea units into one of three lexical categories and summed across the two stories: proper names (*n* = 9), verbs (*n* = 14), and numerical expressions (*n* = 4; from here on, referred to as “numbers”). All other items were characterized as “other” (*n* = 23). Finally, following Bruno et al. (2020), we defined serial position in the following manner: “primacy” consisted of the first eight items in each story, “middle” included the next nine items, and the last eight items were defined as “recency.”

### Difficulty and Discrimination Indices

Item “difficulty” is defined as the proportion of participants who answer an item correctly (Hambleton et al., 1991). The difficulty of each item from Stories A (*n* = 25) and B (*n* = 25) from Logical Memory was calculated by dividing the number of correct responses by the total number of responses (*n* = 50; Crocker and Algina, 1986). A difficulty index between 0.2 and 0.8 is usually considered acceptable (Golden et al., 1984). Item “discrimination” is the extent to which items distinguish between high vs. low performers on the test; item discrimination was calculated by corrected item-total correlations for each item with the remaining items. The acceptable values are 0.2 or higher; the closer to 1, the better the discrimination (Golden et al., 1984). Items with very high or very low difficulty values will therefore often have low discrimination values. For Aim 1, we calculated difficulty and discrimination indices for each item, lexical category, and serial position group for each visit with at least one Logical Memory assessment and used these in analyses described in section “Statistical Analyses.” For Aim 2, we selected the Logical Memory assessment closest to the most recent PET assessment for each person with at least one PET amyloid scan, and we used these values to calculate difficulty and discrimination indices for Aim 2 analyses.

### Molecular Neuroimaging

All participants in the Aim 2 analyses underwent a [^11^C] Pittsburgh compound B (PiB) PET scan on a Siemens EXACT HR+ scanner; PiB processing and quantification methods are described in detail elsewhere (Johnson et al., 2014). A 70-min dynamic acquisition using reference Logan graphical analysis (cerebellum gray matter reference region) was used to estimate the PiB distribution volume ratio (DVR). A previously defined global DVR threshold of >1.19 (Sprecher et al., 2015) was used to dichotomize individuals as amyloid positive or negative (Aβ+/−).

### Statistical Analyses

Participant demographics and clinical characteristics are presented overall, as well as by those with vs. without a PET amyloid scan. In the subset with PET amyloid data, the Aβ+ vs. Aβ− groups are described using tests appropriate for the distribution of the variables (e.g., *t*-tests, chi-square tests, or ANCOVA).

Difficulty and discrimination indices were calculated for each visit as described in “Difficulty and Discrimination Indices” section using “*sjPlot*”.^1^ For Aim 1 analyses testing whether item difficulty or discrimination indices differ by recall condition, we conducted repeated measures ANOVAs of the paired item-level differences (immediate minus delayed recall; separate models for differences in difficulty and discrimination), adjusting for repeated measures across visits. We included a story version group variable to test whether paired differences in immediate to delay difficulty or discrimination indices were the same across story versions A and B. We plotted the item difficulty and discrimination differences (mean across visits and by visits) and qualitatively described which items differ most from immediate to delayed condition.

For analyses examining whether each of the two psychometric indices (difficulty and discrimination) differed by story version, lexical category, or serial position within a recall condition, we ran separate repeated measures ANOVAs for immediate recall and delayed recall difficulty and discrimination. After observing that the residuals of the models failed the normality assumption, we reran the analyses using general linear mixed effect models (R package “glmmTMB”; we used R package “DHARMa” to run residual diagnostics for these models). *Post hoc* analysis (e.g., pairwise comparisons following a significant omnibus test for a group variable with more than two groups) and effect size were calculated by R package “emmeans.”

For Aim 2 analyses testing whether item difficulty or discrimination indices differed by amyloid status, we calculated the item-level difficulty and discrimination indices separately for the Aβ+ and Aβ− groups using the item-level data for the Logical Memory visit closest to the PET PiB scan. To examine whether Aβ+ and Aβ− groups differed in difficulty or discrimination, we used a two-sample *t*-test if the normality and homogeneity of variances assumptions were satisfied; otherwise, a Mann–Whitney U test was used. We followed this procedure for each recall condition, and within recall condition, for each story version, lexical category, and serial position group. For qualitative inspection of differences, we calculated the paired item-level differences in difficulty and discrimination indices between the Aβ+ and Aβ− groups for each item, story version, and recall condition and then used paired *t*-tests or Wilcoxon signed rank tests to test whether items within a subset of items differed in difficulty or discrimination between Aβ+ and Aβ− (item subsets for each recall condition included story version, lexical categories, and serial position groups).

For all models, magnitudes of between-group differences were characterized using Cliff’s delta, which were calculated using the “effsize” package in R (Torchiano and Torchiano, 2020). Cliff’s delta is a non-parametric effect size measure that quantifies the amount of difference between two groups of observations beyond the values of *p* interpretation, which is less susceptible to outliers and skewness than Hedges’ *g* or Cohen’s *d* and better in circumstances where the homogeneity of variance assumption does not hold (Cliff, 1993). The magnitude is assessed using the thresholds provided in Romano et al., (2006), i.e., |d| < 0.147 “negligible,” |d| < 0.33 “small,” |d| < 0.474 “medium,” otherwise “large.” Analyses were performed in R 4.0.2. Significance level was set at *p* < 0.05.

## Results

Participant demographics and clinical characteristics are presented overall for the Aim 1 sample (*n* = 1,141) and overall and by amyloid status for the Aim 2 subsample (*n* = 338) in Table 1. The overall sample had an average age of 58.6 (SD = 6.6) at the first Logical Memory visit, 6% identified as Black or African American, 92% identified as non-Hispanic White, 2% identified as Hispanic, Asian, Native American/Indian, or other; the sample overall had 16 years of education (SD = 2.3).

**Table 1:** Demographic and clinical characteristics by total sample and subsample with amyloid imaging.

	Whole sample	No PET subsample	PET subsample	Amyloid positive (Aβ+)	Amyloid negative (Aβ-)
*n*	1,141	803	338	79	259
Age at Logical Memory baseline	58.55 (6.64)	58.44 (6.68)	58.82 (6.54)	61.05 (4.93)	58.14 (6.82)^#^
Age at most recent visit	65.27 (7.18)	64.57 (7.23)	66.92 (6.79)	69.56 (4.88)	66.11 (7.08)^#^
Age at most recent PET scan			67.58 (7.13)	70.59 (5.14)	66.66 (7.41)
Sex (% female)	800 (70.1)	571 (71.1)	229 (67.8)	53 (67.1)	176 (68.0)
Race (%)					
African-American	67 (5.9)	54 (6.7)	13 (3.8)	3 (3.8)	10 (3.9)
Non-Hispanic White	1,046 (91.7)	727 (90.5)	319 (94.4)	75 (94.9)	244 (94.2)
Other	28 (2.5)	22 (2.7)	6 (1.8)	1 (1.3)	5 (1.9)
Parental history of AD (%)	839 (73.7)	589 (73.4)	250 (74.2)	67 (84.8)	183 (70.9)^#^
WRAT-3 reading standard score	107.46 (9.21)	106.90 (9.52)	108.77 (8.31)^*^	108.97 (7.40)	108.71 (8.58)
Total years of education	15.82 (2.26)	15.70 (2.25)	16.09 (2.25)^*^	16.19 (2.12)	16.07 (2.29)
*APOE-e4* carriers (%)	439 (39.2)	309 (39.2)	130 (39.2)	54 (69.2)	76 (29.9)^#^
CDR or QDRS	0.05 (0.16)	0.06 (0.16)	0.04 (0.13)	0.00 (0.00)	0.04 (0.14)
MMSE	29.39 (0.94)	29.37 (0.96)	29.44 (0.89)	29.44 (0.90)	29.44 (0.88)
R-AVLT total	50.87 (8.57)	50.69 (8.72)	51.30 (8.18)	51.96 (8.54)	51.10 (8.08)
Logical Memory total immediate recall score (range = 0–50)	29.16 (6.23)	28.77 (6.33)	30.07 (5.91)^*^	30.72 (5.77)	29.87 (5.95)
Logical Memory total delayed recall score (range = 0–50)	25.81 (6.96)	25.39 (7.12)	26.80 (6.46)^*^	27.25 (6.68)	26.66 (6.40)
Logical Memory Proper Names Immediate (range 0–9)	6.34 (1.59)	6.30 (1.61)	6.46 (1.53)	6.44 (1.35)	6.46 (1.59)
Logical Memory proper names delayed (range 0–9)	4.89 (2.10)	4.81 (2.15)	5.08 (1.99)	4.99 (2.08)	5.10 (1.96)
Logical Memory verbs immediate (range 0–14)	8.77 (2.28)	8.67 (2.30)	9.03 (2.22)^*^	9.14 (2.21)	9.00 (2.23)
Logical Memory verbs delayed (range 0–14)	8.00 (2.46)	7.91 (2.49)	8.21 (2.36)	8.37 (2.45)	8.17 (2.34)
Logical Memory numbers immediate (range 0–4)	2.64 (1.01)	2.63 (1.02)	2.69 (0.99)	2.78 (0.97)	2.66 (0.99)
Logical Memory numbers delayed (range 0–4)	2.49 (1.08)	2.47 (1.08)	2.53 (1.07)	2.61 (1.07)	2.50 (1.07)
Logical Memory others immediate (range 0–20)	10.78 (2.87)	10.59 (2.88)	11.24 (2.81)^*^	11.72 (2.79)	11.10 (2.81)
Logical Memory others delayed (range 0–20)	9.89 (2.98)	9.68 (2.99)	10.41 (2.90)^*^	10.75 (3.00)	10.30 (2.87)

WRAT-3, Wide Range Achievement Test-3 Reading Subtest (Wilkinson, 1993); MMSE, Mini-Mental Status Examination (Folstein et al., 1975); R-AVLT, Rey Auditory Verbal Learning Test (Schmidt, 1996); and Logical Memory, subtest from the Wechsler Memory Scale-Revised (WMS-R; Wechsler, 1987). PET, Positron Emission Tomography; CDR, Clinical Dementia Rating Scale (Morris, 1997); QDRS, Quick Dementia Rating System (Galvin, 2015); APOE-e4, Apoliopoprotein, allele 4; *t*-tests, chi-square tests, and Mann–Whitney U tests used, depending on distribution.

* Indicates column 2 vs. column 3 statistical significance at *p* < 0.05.

# Indicates column 4 vs. 5 statistical significance at *p* < 0.05.

### Aim 1: Difficulty and Discrimination Indices in the Full Sample

#### Difficulty Indices and Differences Between Recall Condition

**Figure 2:**
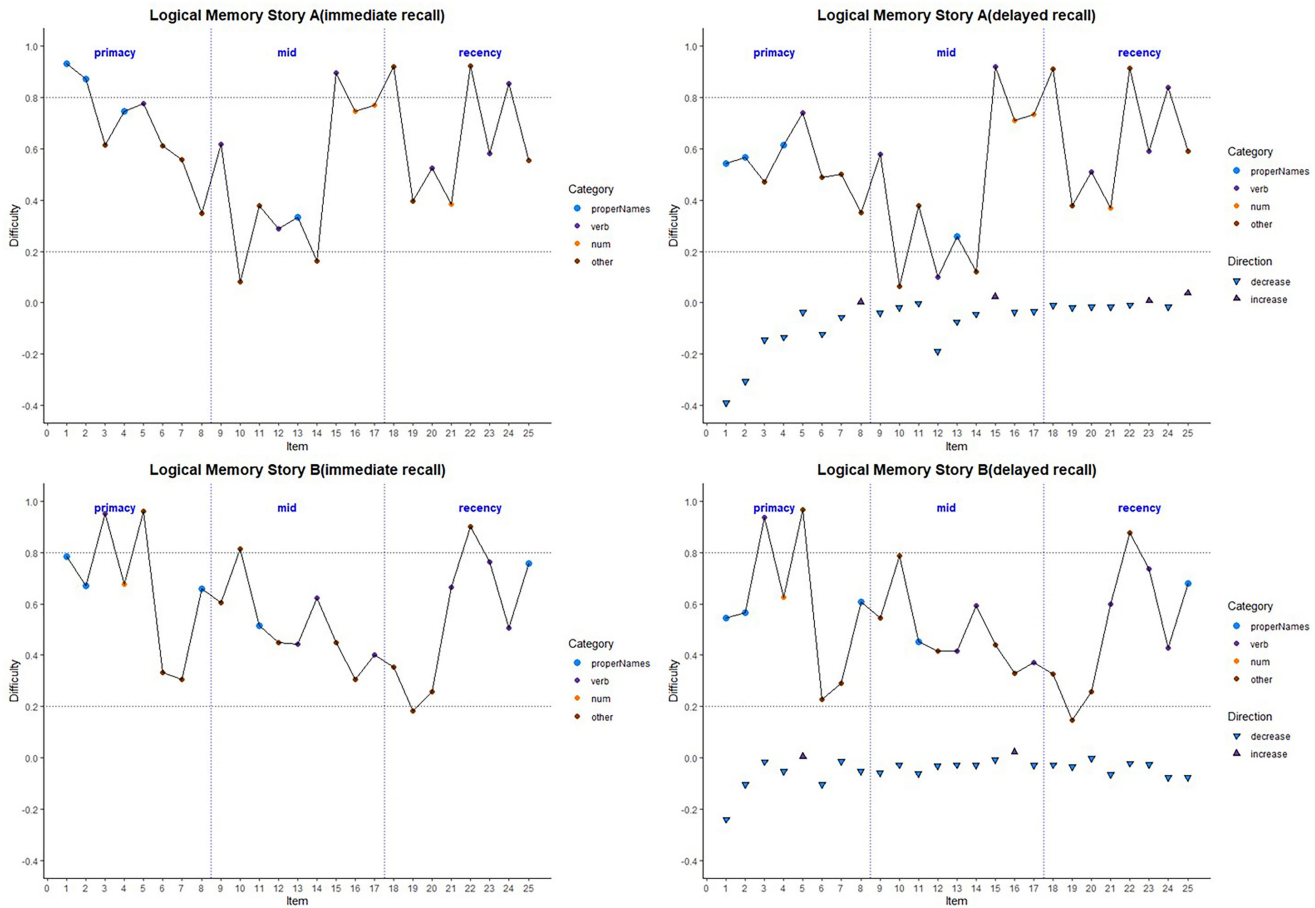
Item difficulty plots (averaged across visits) according to the serial position (primacy, mid, and recency) as well as the lexical category of the items, by story A and story B. Across the primacy, mid, and recency positions, proper name recall shows a drop in percent correct (increase in difficulty) for both story A and story B. The triangles in the right-hand panels are the mean delayed condition percent correct minus mean immediate percent correct for story A and story B. The horizontal dashed lines are desirable difficulty values (between 0.2 and 0.8). Supplementary Figure S1 shows item difficulties by visit, revealing a consistent pattern across all study visits.

Item-level mean difficulty indices across visits for Stories A and B are presented in Figure 2 by immediate (left) and delayed recall (right); colored circles indicate lexical categories, and vertical dotted lines delineate serial position subgroups (Supplementary Figure S1 shows the same, by visit). The triangles in the right-hand panel represent the difference in percent correct between immediate and delayed recall for each item; negative values indicate increased difficulty for delayed relative to immediate recall condition. Qualitatively, items 1 and 2 show the largest drops in proportion correct within each story (i.e., showed the largest increase in item difficulty from immediate to delayed recall). Mean(SD) change in difficulty between immediate and delayed recall was 0.056(0.08), indicating a significant increase in difficulty at delayed recall (generalized linear mixed model adjusting for multiple visits, intercept beta = 0.56; *p* < 0.001). The change in difficulty between recall conditions did not differ between stories A and B (story version beta = −0.01; *p* = 0.39).

#### Difficulty Indices: Differences Within Recall Condition Between Story, Serial Position, and Lexical Category

**Figure 3:**
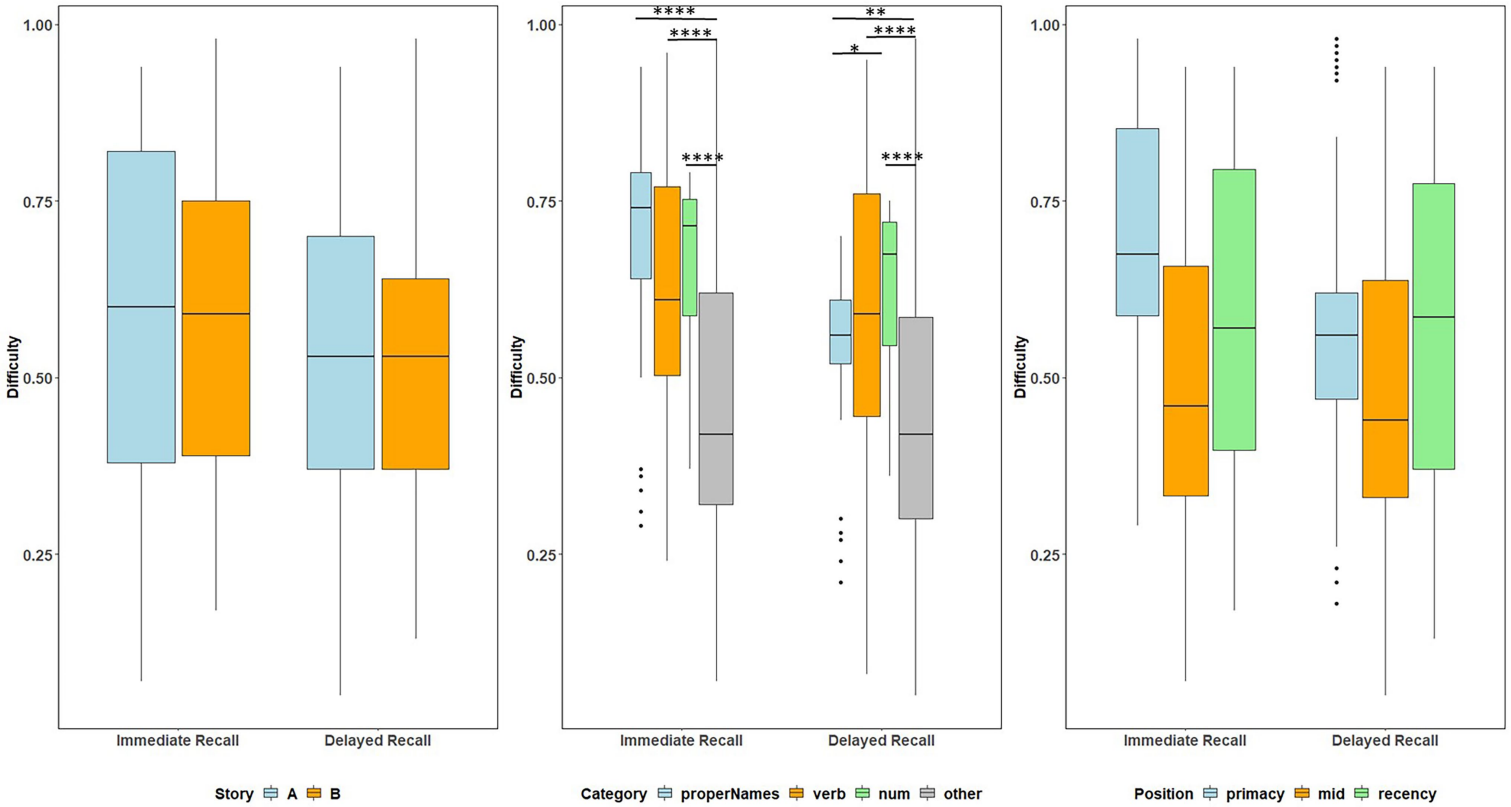
Item difficulty plots at all visits according to the story (A and B), serial position (primacy, mid, and recency) as well as the lexical category (proper names, verbs, numbers, and others) of the items, by immediate recall and delayed recall. The corresponding model information is in Table 2. The Y-axis values represent proportion correct (and thus, lower values indicate more difficult items). *Post hoc* pairwise group differences at unadjusted *p* < 0.05 noted as *< 0.05, **< 0.01, ***<0.001, and ****<0.0001.

Boxplots of item difficulties are shown separately for immediate and delayed recall conditions in Figure 3 by story (left), lexical category (middle), and serial position group (right). GLMM’s showed that lexical category was a significant predictor of difficulty for both immediate and delayed recall conditions (*p* < 0.0001; Table 2); serial position group and story version were not significant predictors in either recall condition. Boxplots of item difficulties (Figure 3) depict across-visit mean difficulties by story version, lexical category, and serial position. *Post hoc* pairwise differences between lexical categories showed significantly lower proportions correct in the “Other” category compared to each of the other lexical categories at both immediate and delayed recall. At delayed recall, proper names were significantly more difficult than Numerical Expressions (Table 2; Figure 3).

**Table 2:** GLMM with the difficulty indices for immediate recall and delayed recall predicted by story, lexical category, and serial position.

		Estimate	CI	*p*	*Post hoc*
Immediate recall	Intercept	0.77	0.64–0.90	<0.0001	
	Story B (reference group = Story A)	−0.01	−0.05–0.03	0.567	
	Lexical category (reference group = PN)			<0.0001	PN vs. other (p < 0.0001)
	Verb	−0.02	−0.11–0.08		Verb vs. other (p < 0.0001)
	Num	0.04	−0.07–0.15		Num vs. other (p < 0.0001)
	Other	−0.20	−0.29–−0.12		
	Serial position (reference group = primacy)			0.065	
	Mid	−0.18	−0.33–−0.03		
	Recency	−0.06	−0.22–0.10		
Delayed recall	Intercept	0.58	0.45–0.72	<0.0001	
	Story B	0.01	−0.03–0.05	0.583	
	Lexical category			<0.0001	PN vs. other (*p* = 0.008)
	Verb	0.06	−0.04–0.15		Verb vs. other (p < 0.0001)
	Num	0.13	0.01–0.24		Num vs. other (p < 0.0001)
	Other	−0.12	−0.21–−0.03		PN vs. Num (*p* = 0.036)
	Serial position			0.190	
	Mid	−0.13	−0.29–0.03		
	Recency	0.0022	−0.16–0.17		

Model: generalized linear mixed models were run for immediate recall and delayed recall separately. Item difficulty indices ~ story + lexical category + serial position + repeated measure time + random effects (random item-level intercepts and repeated measurement slopes). Reference group for story version = Story A; Reference group for lexical category = proper names; reference group for serial position = primacy. *Post hoc* pairwise group differences at unadjusted *p* < 0.05 are noted in the right-hand column. For example, PN vs. other indicates proper names differed from other categories in pairwise comparisons. PN, proper names and Num, numbers.

#### Item Level Discrimination Indices and Differences Between Recall Condition

**Figure 4:**
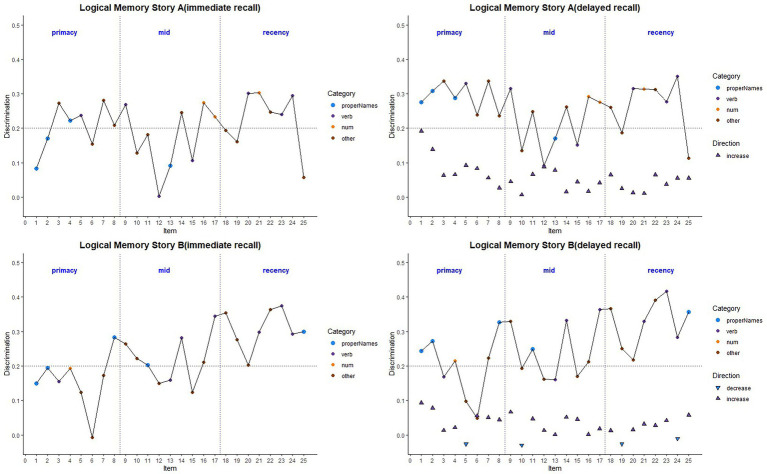
Item discrimination plots (averaged across visits) according to the serial position (primacy, mid, and recency) as well as the lexical category of the items, by story A and story B. Higher discrimination values = better discrimination. Across the primacy, mid and recency positions, proper name recall shows an increase in discrimination for both story A and story B. The triangles are the mean difference between recall condition for story A and story B. The horizontal dashed lines are desirable discrimination values (>0.2). Supplementary Figure S2 shows item discrimination by visit, revealing a consistent pattern across all study visits.

Item-level mean discrimination indices across visits for Stories A and B are presented in Figure 4 by immediate (left) and delayed recall (right); colored circles indicate lexical categories and vertical dotted lines delineate serial position subgroups (Supplementary Figure S2 shows the same, by visit). The triangles in the right-hand panel represent the difference in discrimination indices between immediate and delayed recall for each item; positive values indicate increased discrimination for delayed relative to immediate recall condition. Qualitatively, all story A items, and most story B items show an increase in discrimination for the delayed recall condition. Mean(SD) change in discrimination indices between immediate and delayed recall was 0.043(0.05), indicating a significant increase in discrimination at delayed recall (generalized linear mixed model adjusting for multiple visits, intercept beta = 0.22; *p* < 0.001). The change in discrimination between recall conditions did differ between stories A and B (story version beta = 0.01; *p* = 0.04), indicating a significant increase in discrimination at story B delayed recall.

#### Discrimination Indices: Differences Within Recall Condition Between Story, Serial Position, and Lexical Category

**Figure 5:**
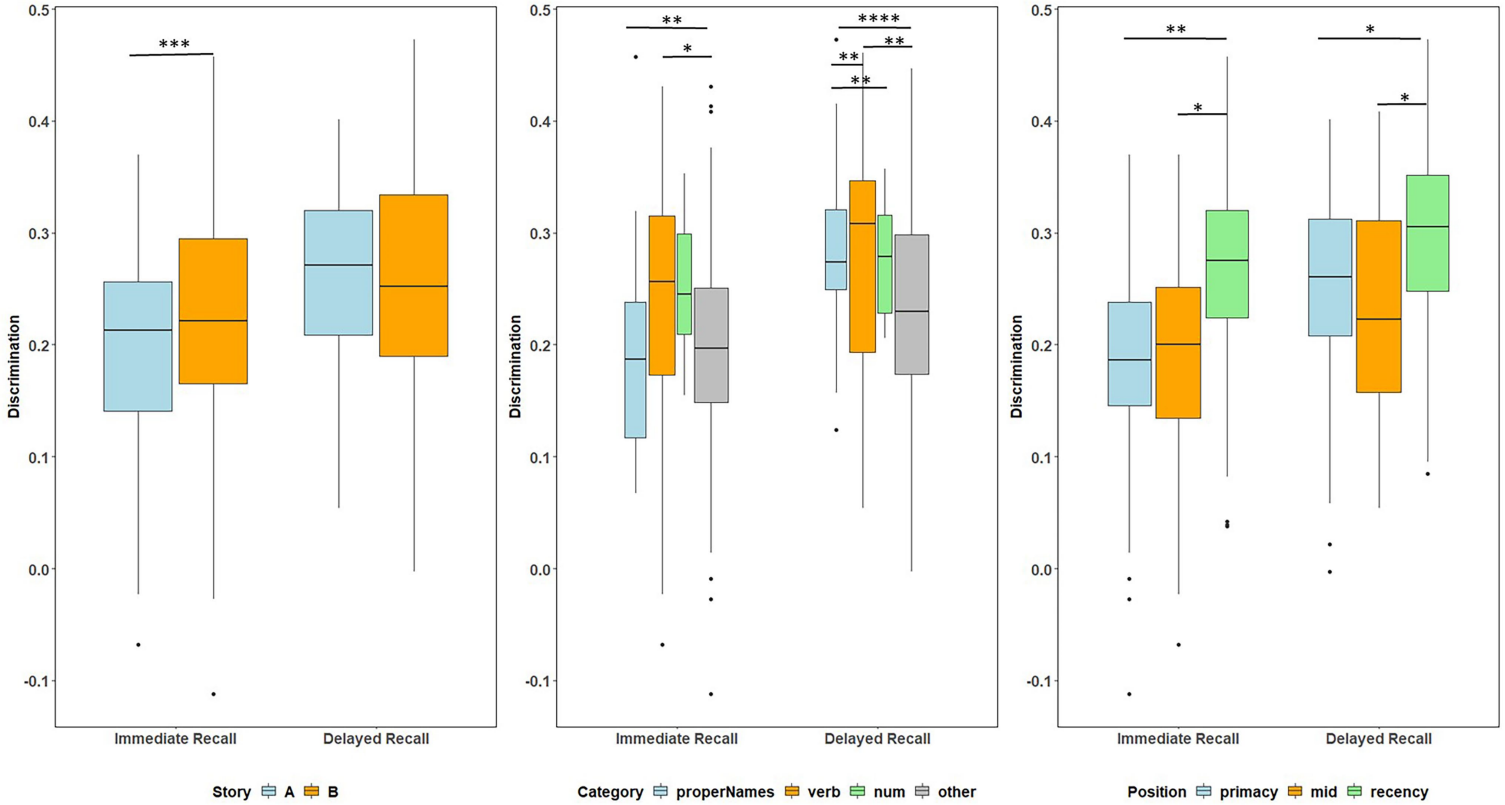
Item discrimination plots at all visits according to the story (A and B), serial position (primacy, mid, and recency) as well as the lexical category (proper names, verbs, numbers, and others) of the items, by immediate recall and delayed recall. The corresponding model information is in Table 3. *Post hoc* pairwise group differences at unadjusted p < 0.05 noted as *< 0.05, **< 0.01, ***<0.001, and ****<0.0001.

Boxplots of item discrimination indices are shown separately for immediate and delayed recall conditions in Figure 5 by story (left), lexical category (middle) and serial position group (right). GLMM’s showed that lexical category was a significant predictor of discrimination for both Immediate and delayed recall conditions (*p* = 0.012 and *p* < 0.0001 respectively; Table 3); serial position group were also significant predictors in immediate (*p* = 0.006) and delayed recall conditions (*p* = 0.027); story version was a significant predictor in immediate recall condition only (*p* < 0.001). Boxplots of item discrimination (Figure 5) depict across-visit mean discriminations by story version, lexical category, and serial position. *Post hoc* pairwise differences between story versions showed significantly higher discriminations in story B at immediate recall, the differences between lexical categories showed lower discriminations in PNs at delayed recall compared to each of the other categories. At immediate recall, PNs discriminated a bit less than the “other” category, too. Verbs had higher discriminations compared to “other” category, and the recency serial position had higher discriminations compared to primacy and mid position at both immediate and delayed recall (Table 3; Figure 5).

**Table 3:** GLMM with the discrimination indices for immediate recall and delayed recall predicted by story, lexical category and serial position.

		Estimate	CI	*p*	*Post hoc*
Immediate recall	Intercept	0.19	0.14–0.24	<0.0001	
	Story B (reference group = Story A)	0.03	0.01–0.05	<0.001	
	Lexical category (reference group = PN)			0.012	PN vs. other (*p* = 0.004)
	Verb	−0.02	−0.06 – 0.01		Verb vs. other (*p* = 0.033)
	Num	−0.02	−0.07 – 0.03		
	Other	−0.05	−0.09 – −0.02		
	Serial position (reference group = Primacy)			0.0055	Primacy vs. recency (*p* = 0.003)
	Mid	0.02	−0.04 – 0.08		Mid vs. recency (*p* = 0.010)
	Recency	0.10	0.03–0.17		
Delayed recall	Intercept	0.28	0.23–0.33	<0.0001	
	Story B	−0.0034	−0.02 – 0.01	0.67	PN vs. other (p < 0.0001)
	Lexical category			<0.0001	Verb vs. other (p = 0.0059)
	Verb	−0.05	−0.09 – −0.01		PN vs. verb (*p* = 0.0089)
	Num	−0.07	−0.11 – −0.02		PN vs. num (*p* = 0.0056)
	Other	−0.09	−0.12 – −0.05		
	Serial position			0.027	Primacy vs. recency (*p* = 0.024)
	Mid	0.00026	−0.06 – 0.06		Mid vs. recency (*p* = 0.018)
	Recency	0.07	0.01–0.13		

Model: generalized linear mixed model were run for immediate recall and delayed recall separately. Item discrimination indices ~ story + lexical category + serial position + repeated measure time + random effects (random item-level intercepts and repeated measurement slopes). Story A, lexical category proper names, and serial position primacy are reference levels. *Post hoc* pairwise group differences at unadjusted *p* < 0.05 noted in right-hand column. For example, PN vs. other indicates proper names differed from other category in pairwise comparisons. PN, proper names and Num, numbers.

### Aim 2: Difficulty and Discrimination Indices in PET Subsample

Table 2 shows demographic and clinical characteristics stratified by those individuals who completed PET amyloid scans (*n* = 338) vs. those who did not (*n* = 803), as well as by Aβ+ (*n* = 79, 23%) and Aβ− (*n* = 259, 77%). Those participants who completed a PET scan had significantly higher WRAT-3 reading standard scores (109 vs. 107), reported more education, and had higher baseline Logical Memory total scores (immediate and delayed) than those who did not complete PET scans. Relative to the Aβ− group, the Aβ+ group was significantly older at Logical Memory baseline (61 vs. 58), had a higher percentage of parental history of AD (85% vs. 71%), and had more *APOE-ε4* carriers (69% vs. 30%). Aβ+ did not differ from Aβ− on any of the cognitive measures at baseline.

#### Difficulty Indices

**Figure 6:**
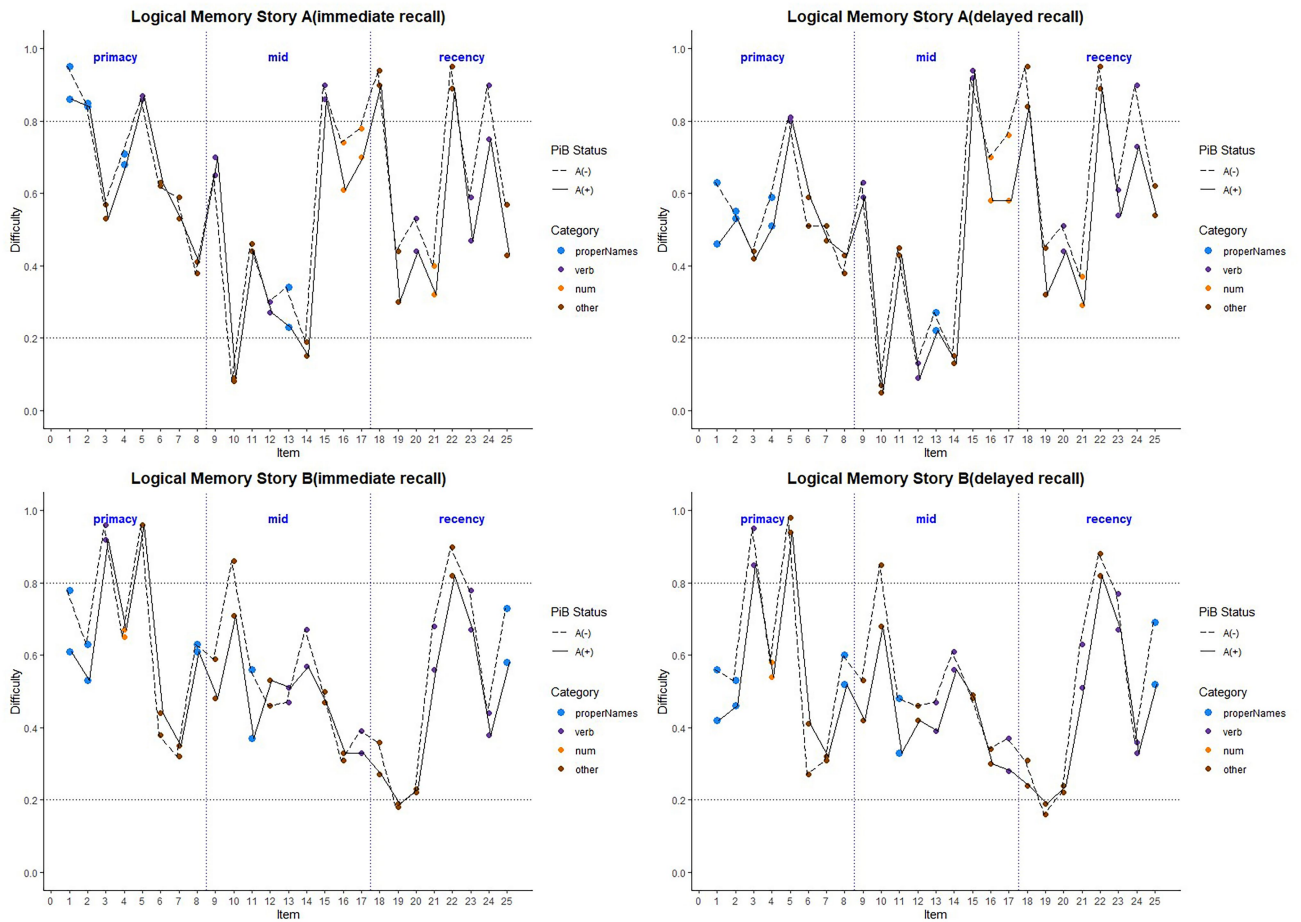
Item difficulty plots by amyloid status according to the serial position (primacy, mid, and recency) as well as the lexical category of the items, by story A and story B. The colored circles indicate lexical categories, vertical dotted lines delineate serial position subgroups, and line types are Aβ+ and Aβ− groups. The horizontal dashed lines are desirable difficulty values (between 0.2 and 0.8). Overall, the mean(SD) immediate recall difficulty was 0.540(0.22) for the Aβ+ group compared with 0.594(0.23) in the Aβ− group (*w* = 1425.5; *p* = 0.24; Cliff’s delta = 0.14). The mean(SD) delayed recall difficulty was 0.485(0.21) for the Aβ+ group compared with 0.545(0.24) in the Aβ− group (*w* = 1466.5; *p* = 0.14; Cliff’s delta = 0.17).

**Figure 7:**
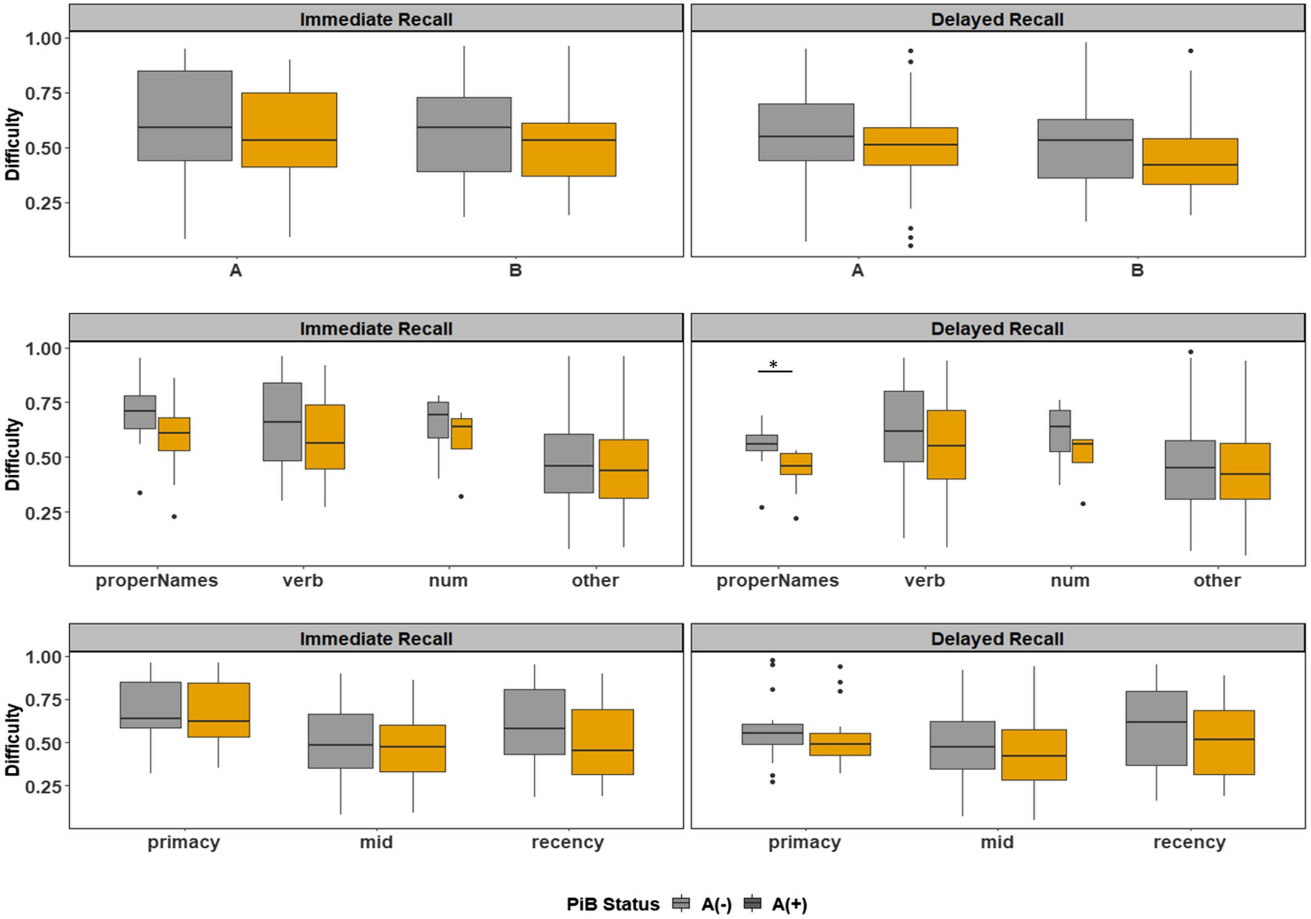
Item difficulty plots by amyloid status according to the story (A and B), serial position (primacy, mid, and recency) as well as the lexical category of the items, by immediate recall and delayed recall. *< 0.05, **< 0.01, ***<0.001, and ****<0.0001.

Figure 6 depicts the difficulty indices by Aβ+ vs. Aβ− for the Logical Memory closest to each person’s last PET scan by story (top = story A and bottom = story B) and recall condition (left = Immediate and right = delayed). Boxplots of item difficulty indices are shown separately for immediate (left) and delayed recall (right) conditions in Figure 7 by story (top), lexical category (middle), and serial position group (below). Descriptive statistics for paired t tests or Wilcoxon signed rank tests are summarized in Table 4; briefly, the difficulty indices of Aβ+ and Aβ− are significantly different in proper names in delayed recall (large Cliff’s delta effect sizes), but not in story versions, other lexical categories, and serial positions both in immediate recall and delayed recall (negligible or small effect sizes).

**Table 4:** The difficulty indices difference between Aβ+ and Aβ− group for immediate recall and delayed recall by story, lexical category, and serial position.

		Aβ+ Mean(SD)	Aβ− Mean(SD)	T Statistic	*p*	Cliff’s delta^a^
Immediate recall	Story A	0.556(0.25)	0.612(0.25)	−0.795	0.43	−0.14
	Story B	0.524(0.20)	0.576(0.22)	−0.879	0.38	−0.14
	Lexical category					
	Proper names	0.590(0.20)	0.687(0.18)	−1.081	0.30	−0.33
	Verb	0.593(0.21)	0.651(0.21)	−0.743	0.46	−0.16
	Num	0.575(0.17)	0.643(0.17)	−0.55	0.60	−0.38
	Other	0.482(0.24)	0.514(0.26)	−0.437	0.66	−0.08
	Serial position					
	Primacy	0.652(0.19)	0.678(0.21)	−0.35	0.72	−0.10
	Mid	0.464(0.21)	0.514(0.23)	−0.687	0.50	−0.11
	Recency	0.512(0.24)	0.601(0.25)	−1.023	0.31	−0.23
Delayed recall	Story A	0.496(0.24)	0.554(0.25)	−0.849	0.40	−0.17
	Story B	0.474(0.20)	0.536(0.23)	−1.047	0.30	−0.19
	Lexical category					
	Proper names	0.441(0.11)	0.544(0.12)	68.5*	0.015	−0.69
	Verb	0.551(0.24)	0.619(0.24)	−0.756	0.457	−0.19
	Num	0.498(0.14)	0.602(0.17)	12*	0.30	−0.50
	Other	0.460(0.24)	0.490(0.27)	−0.41	0.68	−0.10
	Serial position					
	Primacy	0.542(0.17)	0.575(0.20)	154*	0.34	−0.20
	Mid	0.415(0.22)	0.482(0.24)	−0.869	0.39	−0.19
	Recency	0.507(0.23)	0.586(0.26)	−0.915	0.37	−0.19

* Statistical tests: Wilcoxon signed rank tests were performed when both Aβ+ and Aβ− are not approximately normally distributed or do not have approximately the same variance.

a The magnitude is assessed using the thresholds provided in Romano et al. (2006), i.e., |d| < 0.147 “negligible,” |d| < 0.33 “small,” |d| < 0.474 “medium,” and otherwise “large.”

#### Discrimination Indices

**Figure 8:**
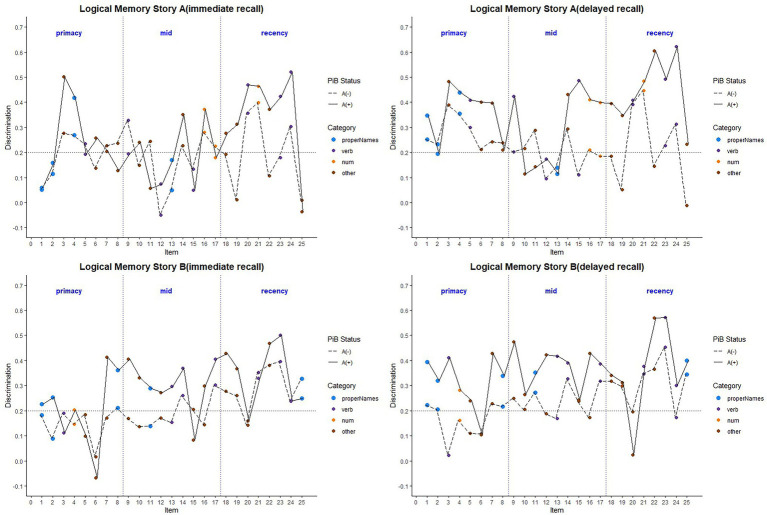
Item discrimination plots according to the serial position (primacy, mid, and recency) as well as the lexical category of the items, by story A and story B. The colored circles indicate lexical categories, vertical dotted lines delineate serial position subgroups and line types are Aβ+ and Aβ− group. The horizontal dashed lines are desirable discrimination values (>0.2). For immediate recall, the mean(SD) discrimination index was 0.540(0.22) for the Aβ+ group compared with 0.594(0.23) in the Aβ− group (*w* = 850.5; *p* = 0.0059; Cliff’s delta = −0.32). For delayed recall, discrimination was 0.485(0.21) for the Aβ+ group compared with 0.545(0.24) in the Aβ− group (*w* = 530.5; *p* < 0.0001; Cliff’s delta = −0.58).

**Figure 9:**
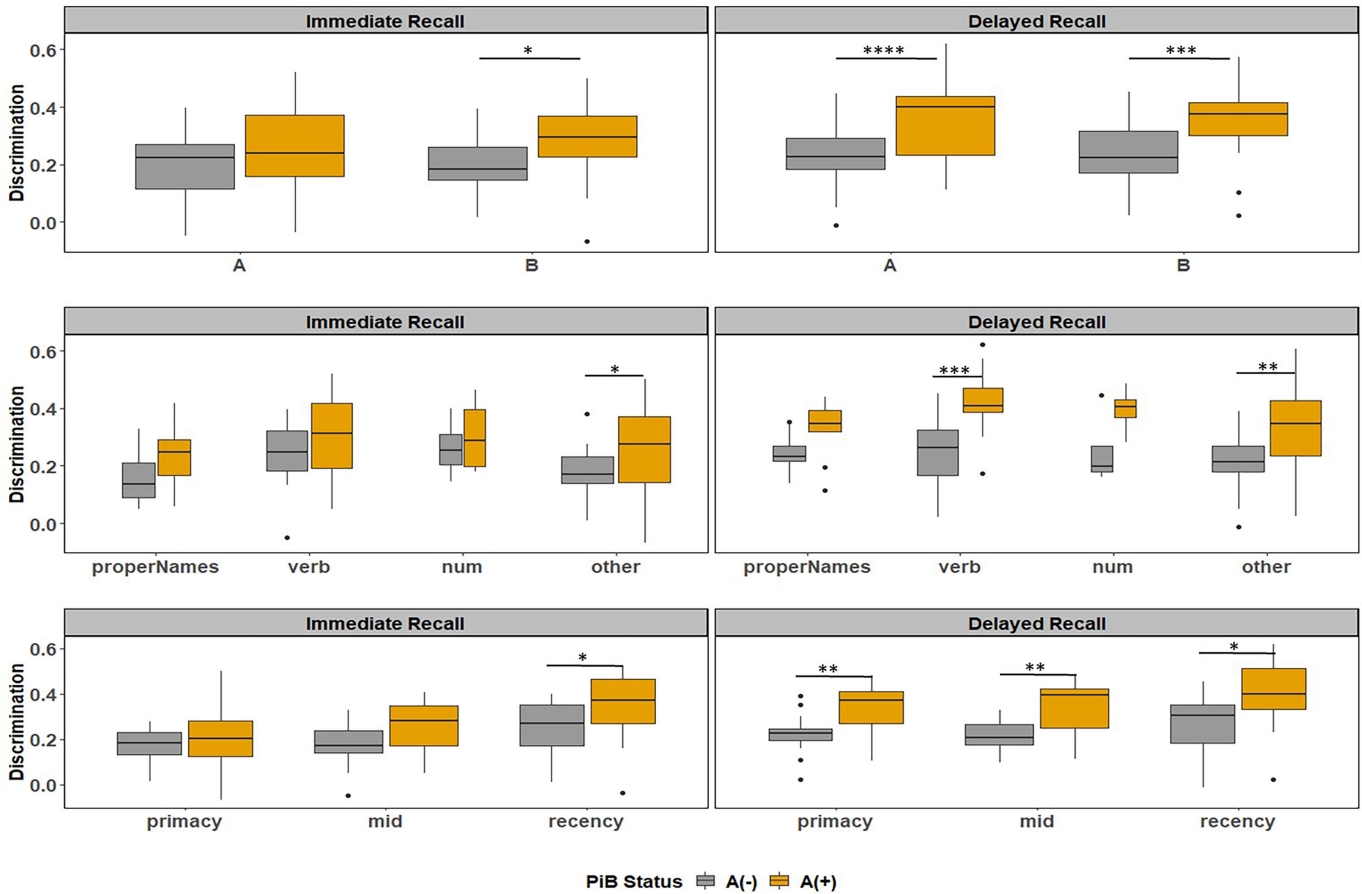
Item discrimination plots by amyloid status according to the story (A and B), serial position (primacy, mid, and recency) as well as the lexical category of the items, by immediate recall and delayed recall. *< 0.05, **< 0.01, ***<0.001, and ****<0.0001.

Figure 8 depicts the discrimination indices for the Logical Memory closest to each person’s last PET scan by story (top = story A and bottom = story B) and recall condition (left = Immediate; right = delayed). Boxplots of item discrimination indices are shown separately for immediate (left) and delayed recall (right) conditions in Figure 9 by Story (top), lexical category (middle) and serial position group (bottom). Descriptive statistics for paired t tests or Wilcoxon signed rank tests are summarized in Table 5; briefly, the discrimination indices differed between Aβ+ and Aβ− by story versions, proper names, “other” lexical categories, and all serial positions, with large or medium Cliff’s delta effect sizes.

**Table 5:** The discrimination indices difference between Aβ+ and Aβ− group for immediate recall and delayed recall by story, lexical category, and serial position.

		Aβ+ Mean(SD)	Aβ− Mean(SD)	*T* Statistic	*p*	Cliff’s delta^a^
Immediate recall	Story A	0.256(0.16)	0.188(0.12)	1.758	0.086	0.25
	Story B	0.284(0.13)	0.21(0.09)	2.279	0.028	0.40
	Lexical category					
	Proper names	0.243(0.11)	0.159(0.10)	1.737	0.10	0.46
	Verb	0.298(0.16)	0.241(0.12)	1.08	0.29	0.24
	Num	0.305(0.14)	0.262(0.11)	0.49	0.64	0.13
	Other	0.258(0.16)	0.177(0.09)	2.13	0.04	0.36
	Serial position					
	Primacy	0.220(0.15)	0.171(0.08)	104.5*	0.39	0.18
	Mid	0.247(0.12)	0.182(0.09)	1.823	0.078	0.36
	Recency	0.346(0.15)	0.246(0.13)	2.07	0.047	0.45
Delayed recall	Story A	0.367(0.14)	0.228(0.11)	3.869	0.00035	0.54
	Story B	0.351(0.12)	0.236(0.10)	3.729	0.00053	0.60
	Lexical category					
	Proper names	0.322(0.10)	0.249(0.07)	1.779	0.097	0.43
	Verb	0.419(0.11)	0.246(0.12)	3.933	0.00057	0.73
	Num	0.394(0.08)	0.251(0.13)	1.83	0.13	0.63
	Other	0.331(0.15)	0.214(0.09)	3.149	0.0032	0.50
	Serial position					
	Primacy	0.337(0.11)	0.218(0.09)	3.431	0.0018	0.59
	Mid	0.337(0.13)	0.215(0.07)	71^*^	0.0042	0.56
	Recency	0.405(0.16)	0.265(0.14)	2.728	0.011	0.54

* Statistical tests: Wilcoxon signed rank tests were performed when both Aβ+ and Aβ− are not approximately normally distributed or do not have approximately the same variance.

a The magnitude is assessed using the thresholds provided in Romano et al. (2006), i.e., |d| < 0.147 “negligible,” |d| < 0.33 “small,” |d| < 0.474 “medium,” and otherwise “large.”

## Discussion

The current study investigated the item-level difficulty and discrimination indices from a classic widely used neuropsychological measure to assess episodic memory function, the Logical Memory story recall task from the Wechsler Memory Scale—Revised (Wechsler, 1987). This test was first published in 1945, with revisions in 1987, 1997, and 2009, thus we draw attention to its longevity and long-standing usage in the field of neuropsychology, aging, and cognitive disorders. The indices were calculated for two story versions, A and B, and for the immediate and delayed recall conditions. We further examined items by other process scores, including the lexical categories to which the items belonged (proper names, verbs, and numerical expressions) and the serial position in which the items were presented. Finally, we evaluated the degree to which the process score groupings differed in their difficulty and discrimination between amyloid positive and negative groups. It was anticipated that item difficulty and discrimination would vary by position in the story (serial position) and/or the lexical category to which the item belonged (e.g., proper names and verbs), as well as by amyloid status.

In a large sample with longitudinal Logical Memory data, item difficulty dropped (i.e., became more difficult) by an average of 10% from the immediate to delayed recall across both story A and story B. This drop did not differ between the two story versions. Poorer delayed recall vs. immediate recall is an unsurprising finding, given that the delayed recall of Logical Memory and other learning tasks such as the Auditory Verbal Learning Test (AVLT) have been shown to be sensitive to MCI and dementia, and are included in widely utilized composite scores (Donohue et al., 2014; Knopman et al., 2019). Although several studies have demonstrated that list learning tasks such as AVLT are more sensitive to decline than story recall (Weissberger et al., 2017), the item-level approach we show here may spur renewed interest in evaluating existing measures or implementing new story recall tasks in future AD studies. Because AD treatments are most likely to be beneficial at the earliest stage of disease, it is important to develop more sensitive measures of cognitive decline for clinical trials (Snyder et al., 2014). The Federal Drug Administration has indicated the need for improved outcomes for AD clinical trials, not only for those that are more sensitive to change, but also for those that measure functional abilities (U.S. Department of Health and Human Services, 2018). Story recall tasks have an element of ecological validity that learning a list of 10 unrelated items does not. By developing new story recall scoring metrics or tasks that weigh semantic/lexical properties, serial position, and item difficulty and discrimination, we may be able to increase sensitivity to AD-related cognitive decline, while maximizing an ecologically valid task.

Our findings also highlight that there was no difference in delayed recall item difficulty between story A and story B. Previous studies examining alternate forms of story recall have shown similar diagnostic sensitivity to one another (Cunje et al., 2007). To our knowledge, our study is the first to empirically confirm the similarity in difficulty of items for story A and story B of Logical Memory delayed recall. This finding is important, because many worldwide AD studies are utilizing Logical Memory, administering only Story A, only story B, or both (Toga et al., 2016). Therefore, this empirically derived information may be useful for other studies utilizing (or planning to implement) various forms of Logical Memory in longitudinal, aging cohorts. Moreover, the results presented here offer support for the prospect of using Story A and Story B as alternate versions of one another in a test–retest scenario.

Item difficulty on immediate recall differed between lexical categories, with the “other” category being more difficult than the other three lexical categories (proper names, verbs, and numerical expressions) on both recall conditions. This may relate to the fact that many of the items in the “other” category are less concrete (i.e., imageable), than proper names, nouns, and verbs; for example, the idea unit “the night before” presents as more difficult than the idea unit/verb “robbed.” Furthermore, some of the items with the highest emotional valence tended to be verbs (“had not eaten”); abundant evidence indicates that individuals tend to encode items with emotional valence over those without (Kensinger and Corkin, 2004; Thomas and Hasher, 2006; Satler et al., 2007; Petrican et al., 2008).

We did not see overall differences in item difficulty by their position in the stories, in either immediate or delayed recall. However, there was higher discrimination for items in the recency position as compared to the middle and primacy positions in both the immediate and delayed recall conditions. In other words, more recent items were better discriminated among ability levels than items in the primacy or middle positions. The typical pattern in list learning tasks is that performance is better for stimuli learned at the beginning (primacy) or at the end (recency), as compared with items in the middle (Murdock, 1962), while individuals with mild cognitive impairment or dementia tend to show a pronounced deficit at the recency position when comparing immediate to delayed recall conditions (Carlesimo et al., 1995; Bruno et al., 2016, 2018). The fact that our analyses showed that items in the recency position were best at discriminating between ability levels may reflect differences in underlying cognitive abilities (or decline in abilities) in this at-risk cohort.

Item discrimination was higher at delayed than the immediate recall condition, with Story B having a significantly higher discrimination than Story A. On immediate recall, average item discrimination was higher for Story B compared to A; for “other” compared to proper names. On delayed recall, proper names had better discrimination than each of the other lexical categories. Proper name recall in conversation is a common complaint of older individuals (Burke et al., 1991; Gollan et al., 2005; van Harten et al., 2018), and proper name recall has been shown to decline with age (Maylor and Valentine, 1992; Burke et al., 2004). However, whether there is an age differential in the actual difficulty in learning and recall of proper names vs. other lexical categories in aging is up for debate (Cohen and Faulkner, 1986; Cohen and Burke, 1993; James, 2006). The results of the present study indicate that proper names are better able to discriminate among ability levels than other lexical categories and may provide further evidence for utilizing semantic memory tasks that target proper names for early detection of subtle cognitive decline (Fine et al., 2011; Papp et al., 2014; Rubiño and Andrés, 2018; Alegret et al., 2020).

In the subset with PET amyloid imaging, item-level analyses suggest that all items in the delayed recall condition of Logical Memory (both stories A and B) discriminate well between Aβ+ and Aβ−, which is consistent with reports of the story recall tasks’ sensitivity to stages of cognitive decline and AD pathology, and helps explain why the task is featured in popular AD memory composite scores (Donohue et al., 2014; Knopman et al., 2019). With respect to item difficulty, proper names at delayed recall were significantly more difficult for Aβ+ than Aβ−. This finding is consistent with our previous study showing an association between delayed recall of proper names and amyloid positivity (Mueller et al., 2020). Although most items of both stories in both conditions appear to be more difficult in the Aβ+ group, none of the other lexical categories or any of the serial position difficulty indices were significantly different between the two groups.

Analyses also revealed the items in the verb and “other” lexical categories and all serial positions from delayed recall were more discriminate for the Aβ+ group compared to the Aβ− group. That proper names were not significantly more discriminate than the other lexical categories (but were more difficult) may indicate an earlier “loss” of these items in the Aβ+ group. When applying item response theory to items of the Mini-Mental Status Examination (MMSE; Folstein et al., 1975), Ashford et al. described difficulty as a continuum of ability, and discrimination as how well an item can differentiate between examinees with a range of ability levels. Applying these concepts to the MMSE, difficulty indicates a loss of ability underlying performance, while discrimination is an indicator of how quickly that function is lost, such that high difficulty and low discrimination indicates early loss across a longer range of progression. Items on the MMSE with the highest difficulty and lowest discrimination in that study were the three words at delayed recall (ball, flag, and tree), indicating that delayed memory was the earliest ability lost on the continuum of dementia severity (Ashford et al., 1989). Another item-level analysis of the MMSE-37 in a Spanish speaking population found that language items were among the best at discriminating between groups with dementia and healthy controls (Prieto et al., 2012). Although we did not examine people with dementia, dementia severity, or progression of AD, it is possible that proper name recall is an ability that is particularly vulnerable to early amyloid pathology; future studies can evaluate item sensitivity to estimated age of onset or projected rate of amyloid accumulation using methods developed by our group (Koscik et al., 2020; Betthauser et al., 2021).

Items significantly discriminated between Aβ+ and Aβ− groups, but when comparing amyloid groups using the typical total score from Logical Memory, there were no significant differences [Table 1; mean(SD) Aβ+ = 27(7), Aβ− = 27(6)]. Here, we show that by performing item difficulty and discrimination indices, sensitivity of specific items to Aβ+ may be higher than using the total score alone. By understanding the item’s characteristics and properties, a more sensitive test, or a more sensitive scoring algorithm than total score, can be developed. This approach of utilizing item response theory has been applied toward groups of items from the Mini-Mental Status Examination (Fillenbaum et al., 1994), where sets of four items were able to discriminate among controls, participants with MCI, and those with dementia with high sensitivity and specificity (Fillenbaum et al., 1994). Additionally, item response theory has been used to create new global cognitive function measures from an array of existing measures (Mungas and Reed, 2000; Mungas et al., 2003; Gershon et al., 2010). Because story recall tasks have an ecologically valid component (the task simulates conversations that often need to be recalled later), the development of a more sensitive story that includes types of items that best discriminate among individuals with evidence of AD pathology would make a needed metric for evaluating response to treatment or disease monitoring in clinical trials (Posner et al., 2017).

Strengths of this study include the large sample size, the longitudinal cohort, the subsample with neuroimaging data, and the detailed analysis of item difficulty and discrimination for two different stories of Logical Memory. Further, this is the first study to characterize these indices by amyloid status in a group of cognitively unimpaired individuals.

A limitation of this study is that the lexical categories of the stories are not balanced or equal in scores, which may bias the results. Additionally, the sample is a highly educated (~16 years education), predominantly white (91%), self-selected cohort of individuals at risk for AD; therefore, the results of this work need to be replicated in diverse cohorts to be able to generalize the findings. The number of individuals who are amyloid positive is relatively small compared to those who are amyloid negative (23% positive vs. 77% negative). Although these percentages are representative of the general population at this early stage of AD neuropathological development, i.e., 25%–30% of individuals in this age group are purported to be amyloid positive (Jack et al., 2018), this likely reduces power to detect significant effect sizes. Furthermore, for the amyloid analyses, we selected the Logical Memory test closest to the PET scan for each participant. For the amyloid positive group, the mean difference in time was 1.07 years, for the amyloid negative group, the mean difference was 0.55 years between Logical Memory and PET scan. Although it is unlikely that many participants were on the cusp of amyloid positivity, it is possible that a small number of participants may be very close to the amyloid positivity cutoff. Future analyses that potentially include longitudinal modeling of AD biomarkers may help address this potential confound. Finally, we did not address practice effects in our amyloid models, which may either skew results for some participants, or may miss important differences in others (Jutten et al., 2020). Future analyses will examine whether practice effects vary by amyloid status.

In sum, we provide empirical evidence that both stories of the Logical Memory task are effective at discriminating ability levels, as well as amyloid status, and that individual items vary in difficulty and discrimination by amyloid status, while total scores do not. These results can be informative for the future development of sensitive tasks or composite scores for early detection, disease monitoring, and response to treatment for clinical trials.

## Data Availability Statement

The datasets presented in this article are not readily available because data are available through a data request process. Requests to access the datasets should be directed to https://wrap.wisc.edu/data-requests/.

## Ethics Statement

The studies involving human participants were reviewed and approved by the University of Wisconsin-Madison Internal Review Board. The patients/participants provided their written informed consent to participate in this study.

## Author Contributions

RK, LD, KM, and BH designed the analyses. LD, RK, and KM analyzed the data. SJ, BC, and TB oversaw data collection and data processing. KM, LD, DB, and RK wrote the manuscript. All authors contributed to the article and approved the submitted version.

## Funding

This work was funded by the following grants from the National Institutes of Health: NIH 1R01AG070940, R01 AG021155, R01AG027161, R01-AG054059, NIH P50 AG033514, and NIH U54 HD090256 and the following grant from the Alzheimer’s Association: AARF-19-614533.

## Conflict of Interest

The authors declare that the research was conducted in the absence of any commercial or financial relationships that could be construed as a potential conflict of interest.

## Publisher’s Note

All claims expressed in this article are solely those of the authors and do not necessarily represent those of their affiliated organizations, or those of the publisher, the editors and the reviewers. Any product that may be evaluated in this article, or claim that may be made by its manufacturer, is not guaranteed or endorsed by the publisher.

## Footnotes

^1^ https://cran.r-project.org/web/packages/sjPlot/sjPlot.pdf
