# Supplementary material for: Item-Level Story Recall Predictors of Amyloid-Beta in Late Middle-Aged Adults at Increased Risk for Alzheimer’s Disease
Source: Front Psychol. 2022 Jun 27;13:908651. doi: 10.3389/fpsyg.2022.908651 (PMC9271832; doi:10.3389/fpsyg.2022.908651)
Supplement: Supplementary file 2 [file Image_2.pdf]

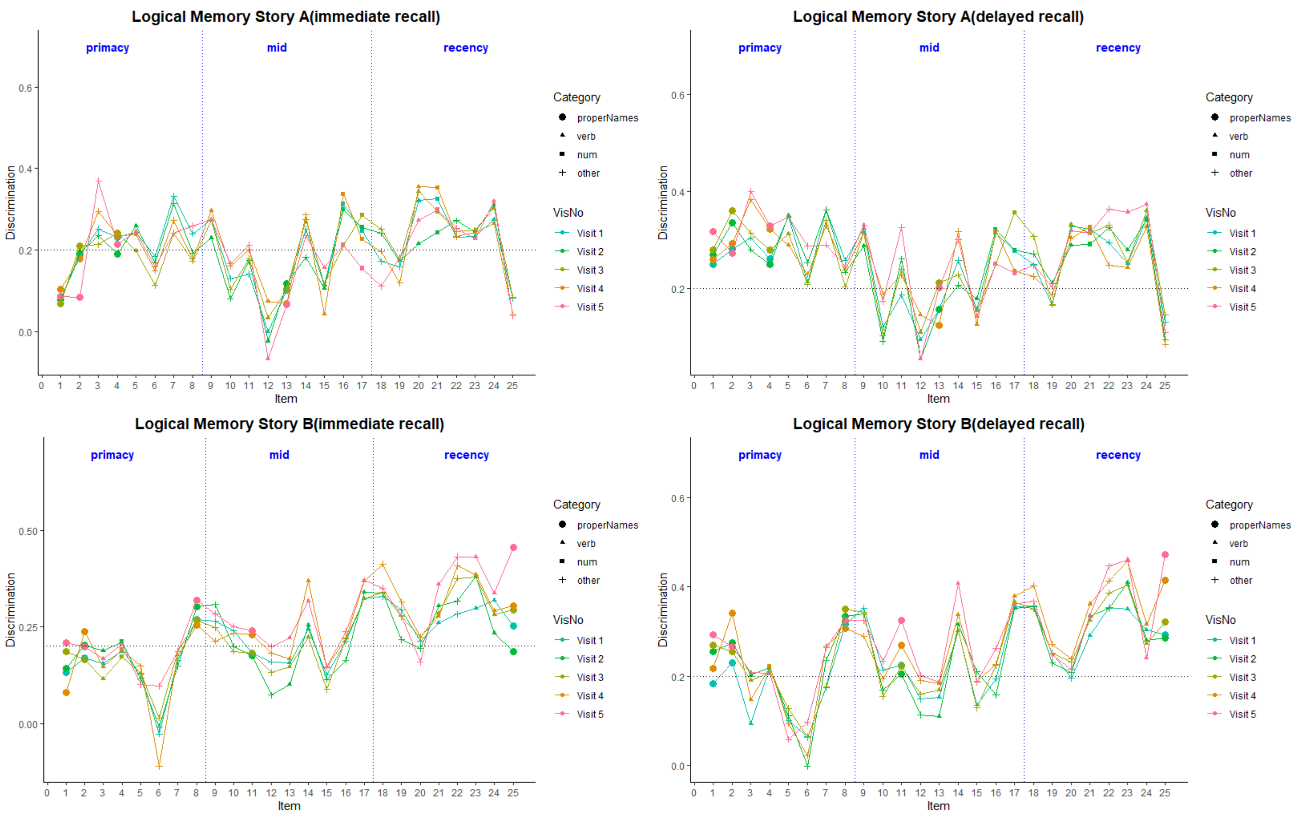

Figure S2. Item level discrimination for Story A and B by serial position, lexical category, and longitudinal visit number
